# Early expression of the Helicase-Like Transcription Factor (HLTF/SMARCA3) in an experimental model of estrogen-induced renal carcinogenesis

**DOI:** 10.1186/1476-4598-5-23

**Published:** 2006-06-08

**Authors:** Gaël Debauve, Denis Nonclercq, Fabrice Ribaucour, Murielle Wiedig, Cécile Gerbaux, Oberdan Leo, Guy Laurent, Fabrice Journé, Alexandra Belayew, Gérard Toubeau

**Affiliations:** 1 Laboratory of Molecular Biology, University of Mons-Hainaut, Mons, Belgium; 2 Laboratory of Histology, University of Mons-Hainaut, Mons, Belgium; 3 Proteomics Unit, Biovallée n.p.c., Gosselies, Belgium; 4 Laboratory of Animal Physiology, Université Libre de Bruxelles, Gosselies, Belgium; 5 Laboratory of Endocrinology and Bone Diseases and Department of Internal Medicine, Institut J Bordet, Brussels, Belgium

## Abstract

**Background:**

The Helicase-Like Transcription Factor (HLTF/SMARCA3) belongs to the family of SWI/SNF proteins that use the energy of ATP hydrolysis to remodel chromatin in a variety of cellular processes. Several SWI/SNF genes are disrupted in cancer, suggesting a role of tumor suppressor. Similarly, the HLTF gene was recently found to be inactivated by hypermethylation in a number of advanced colon and gastric tumors. However, other evidences indicated a 20-fold HLTF overexpression in cell lines derived from various neoplasms (ovary, breast, cervix, kidney...).

**Results:**

In the present study, we investigated HLTF expression by immunohistochemistry in a model of kidney tumors induced by continuous administration of diethylstilbestrol to male Syrian golden hamsters. A strong labeling was already detected in small tumor buds, making HLTF an early cancer marker in this model. Although every cell stained for HLTF at this early stage, the number of HLTF-positive cells decreased to 10% with cancer progression, and these positive cells were dispersed in the tumor mass. HLTF expression was conserved in the HKT-1097 cell line established from kidney tumors, but again only 10% of positive cells were found in xenografts produced by HKT-1097 cells in nude mice.

**Conclusion:**

In conclusion, our data suggest that *HLTF *gene activation is linked to initial steps of carcinogenesis in this model and should be investigated in early stages of other neoplasms.

## Background

The human helicase-like transcription factor (HLTF; SMARCA3 in OMIM) presents a RING-finger motif as well as the 7 DNA helicase domains characterizing the SWI/SNF family of chromatin remodeling factors [1]. Albeit devoid of helicase activity on naked DNA, these proteins are DNA-dependent ATPases that modify chromatin structure. SWI/SNF proteins displace nucleosomes on DNA by the propagation of a DNA bulge, i.e. a local distortion of the DNA double helix wrapped around the histone core (reviewed by [2]). SWI/SNF proteins were initially identified in yeast by the fact that their inactivation caused a global alteration of gene transcription. Some SWI/SNF proteins have been implicated in cellular functions so diverse as chromosome segregation (*lodestar*; [3]), nucleotide excision repair (RAD4, ERCC6, and RAD16; e.g. [4]), nucleosome reassembly after replication (I-SWI; [5]) and DNA double-strand break repair [6]. However, they have been mostly studied for their involvement in the regulation of transcription, where they act as large multiunit complexes (such as RSC, NURF, CHRAC, NURD...; reviewed in [7]).

Most members of the SWI/SNF protein family lack a sequence-specific DNA binding domain and are thought to access promoters by interactions with DNA-bound *trans *elements. In contrast, HLTF can be directly targeted to specific promoters thanks to a specific DNA binding domain. On the basis of this structural feature, HLTF was independently described under different names by several groups that used different target genes, such as the HIV proximal promoter and SV40 enhancers (HIP116; [8]), the human or rat plasminogen activator inhibitor 1 (PAI-1) promoter (HLTF; [1]; P113; [9]), the rabbit uteroglobin promoter (RUSH; [10]), the myosin light chain gene enhancer (Zbu1; [11]), and a *cis *element in the β-globin locus control region (HLTF; [12]). Yet, direct evidences for a role of HLTF in transcription have only been found in the *PAI-1 *gene [1,9,12] and in the β-globin LCR [12]. Two HLTF protein variants were identified. They differed by alternative translation initiation in the same open reading frame (HLTFMet1 and HLTFMet123): transcriptional activity was only shown for the short variant [1,13].

There are indications that HLTF acts as a modulator of cell differentiation during embryogenesis and adult life. In the mouse embryo, HLTF/Zbu1 mRNA and protein were first detected in heart, skeletal muscle and brain at late stages of development (18.5 days pc). Thereafter HLTF expression extended to all postnatal tissues, in parallel with terminal differentiation [11]. The concept that HLTF might participate in the regulation of differentiation is also supported by the time course of its expression during erythrocyte differentiation, and the fact that forced HLTF overexpression results in inappropriate β-globin gene switching in a human leukemia cell line [12].

Many studies have also implicated SWI/SNF genes in cancer (reviewed by [14]. Human tumors of various origins presented inactivating mutations affecting *hSNF5*[15], *SMARCB1/INI1*[16], *RAD54b *[17], *BRG1 *or *hBRM *associated factors [18] genes. The HLTF RING domain is highly similar to the RING domain of BRCA1, a tumor suppressor gene mutated in breast and ovarian cancer. Similarly, the *HLTF *gene was methylated and epigenetically silenced in 30–70% of human colon and gastric cancers, suggesting a role in tumor suppression [19,20]. In contrast, a 20-fold increase in mouse HLTF/Zbu1 mRNA concentration has been observed in several established cell lines of tumor origin (HeLa, MCF7, ...,[11]) suggesting that HLTF over-expression could be associated with neoplastic transformation. However, this does not necessarily indicate that HLTF play a direct role in malignant cell transformation since these cell lines were already transformed.

These conflicting data prompted us to investigate HLTF during malignant transformation and tumor progression. Since the original observation of Kirkman and Bacon [21] that treatment with diethylstilbestrol (DES) provokes the appearance of kidney tumors in male Syrian hamsters, renal neoplasms induced by the continuous administration of natural (i.e. 17β-estradiol) or synthetic (i.e. DES) estrogens to these animals have been extensively utilized in studies pertaining to hormonal and renal carcinogenesis (see reviews in [22,23]). The major advantage of this model lies in the reproducible time-course of tumorigenesis. In DES-treated hamsters, microscopically distinguishable kidney tumors start appearing 6 months after the beginning of drug exposure [24,25]. Tumor formation is preceded by cytological abnormalities and tissue injury which reflect early steps in the chain of events leading to tumorigenesis [24,26-28]. In particular, examination of renal tissue sections from animals exposed to DES for 4–6 months reveals the presence of focal histological abnormalities which may be considered as early signs of neoplastic transformation. Interestingly, these tumorous buds, as well as well-developed kidney malignancies exhibit a variety of lineage markers pointing to a mesenchymal origin (vimentin) and suggesting a relationship with cells of the neuroectodermal lineage (e.g. S100 protein, GFAP). This intriguing finding has led some of us to postulate that DES-induced kidney tumors actually derive from Schwann cells or peripheral nerve sheath cells, although other histological origins cannot be dismissed [25].

A few years ago, primary cultures of kidney tumor samples from hamsters exposed to DES for 11 months resulted in cell immortalization and the emergence of a new established cell line (HKT-1097). HKT-1097 cells express most markers characterizing renal tumors *in vivo *[29,30] and exhibit carcinogenic properties in nude mice (F. Journé, unpublished data).

In the present study, two rabbit antisera raised against individual HLTF protein variants were used for immunocytochemical analyses of DES-induced kidney tumors at various stages of development, and demonstrated HLTF over-expression as an early event. Moreover, this high expression level was maintained in the tumor-derived HKT-1097 cell line.

## Methods

### Cell culture

The major characteristics of the HKT-1097 cell line (ECAAC n° 98061003, DSMZ n° AAC 445) were described in previous publications [29,30]. Cell cultures were maintained at 37°C in a cell incubator with humid atmosphere at 5% CO_2_. Cells were propagated in T75-flasks (Orange Scientific, Braine-l'Alleud, Belgium) containing Dulbecco's Modified Essential Medium (DMEM, BioWhittaker Europe, Verviers, Belgium) supplemented with Phenol Red, 10 % fetal bovine serum (FBS, HyClone, Logan, Utah), 25 mM N-2-hydroxyethylpiperazine-N'-2-ethanesulfonic acid (HEPES), 2 mM L-glutamine, 100 U/ml penicillin G, 100 μg/ml streptomycin, and 0.25 μg/ml amphotericin B (DMEM-FBS) (supplements from BioWhittaker or Gibco-Invitrogen, Merelbeke, Belgium). Cell cultures were passed once a week (split ratio 1:300) and were fed fresh medium every 2–3 days. For routine subculture and cell plating in preparation of immunofluorescence studies, cells were dislodged from the vessel bottom by treatment with trypsin-EDTA solution, suspended in culture medium by vigorous pipetting and counted in a model Z1 Coulter counter.

### HKT-1097 tumor xenograph

HKT-1097 cells were plated at an initial density of 10^4 ^cells/cm^2 ^in 75-cm^2 ^flasks, grown for three days as described above and harvested by trypsinization. After centrifugation the cells were resuspended in serum-free DMEM without Phenol Red, and their concentration adjusted to 5 × 10^7 ^cells/ml. 200 μl aliquots of the cell suspension were injected subcutaneously into the left flank of male nude mice weighing 20–25 g (6 week old) (Swiss strain; Iffa Credo, Brussels, Belgium). Development of the tumor xenograft was monitored weekly. Mice were killed by cervical dislocation and the tumors harvested at necropsy, fixed in Duboscq-Brazil fluid and processed as described above for immunohistochemistry. These experiments had been performed at the Bordet animal facility (accreditation n°LA 1230352) and approved by the Ethics Committee for Animal Welfare of the "Université Libre de Bruxelles".

### Animals and treatment

Male Syrian hamsters (*Mesocricetus auratus*) weighing 70–80 g (3–4 month old) were used throughout the study. The animals were bred and maintained at the UMH animal facility accredited (n°LA 1500021) by the Belgian Ministry of Middle Class and Agriculture, and the experiments performed in compliance with the guidelines edicted by authorities. Five groups of at least 4 animals received chronic treatment with DES following a protocol detailed previously [31,32]. Implants filled with 25 mg of DES (Sigma; S^t ^Louis, MO) were inserted in the subcutaneous tissue of the shoulder area of anesthetized animals and renewed every 2.5 months to maintain a constant blood level of DES (approx. 13 ng/ml). Hamsters (at least 4 animals per time point) were terminated 1, 2, 4, 5, 6, 7, 9, 10 and 11 months after the first implantation. A group of sham-operated animals (n = 4) and a group of untreated animals (n = 4) of various ages (7–9 months) were included as controls.

### Antisera

Two putative immunogenic peptides corresponding to the individual HLTF variants (see Result section) were defined by accessibility prediction programs (Garnier software from the EMBOSS software package; [33]), synthesized at Eurogentec (Seraing, Belgium) and conjugated to a carrier protein (keyhole limpet hemocyanin). Two rabbits were challenged by three intradermal injections of each conjugate at two-week intervals, followed by a booster inoculation one month later. The animals were bled 10 days after the last immunization. The rabbits were housed in the IBMM (Gosselies, Belgium) animal facility according to standard protocols and in agreement with the Animal Care and Use Committee of the "Université Libre de Bruxelles" (n°LA 1500474).

### Transcription/translation assays

*In vitro *transcription/translation assays were carried out in a TNT rabbit reticulocyte lysate (Promega Corporation, Madison, WI, USA) in the presence of T7 RNA polymerase and [^35^S] cysteine (1,000 Ci/mM;Amersham Biosciences, Roosendaal, The Netherlands). The cDNA template used (*pGEM-4Z-HLTF*) has been previously described and encodes both HLTF variants (Ding *et al*., 1996). A luciferase cDNA template was substituted for the HLTF cDNA as a control. The reaction mixtures were boiled in sample buffer (2% SDS, 62.5 mM Tris-HCl pH 6.8, 5% β-mercaptoethanol, 20% glycerol, 0.5% bromophenol blue, 18 mM DTE) and resolved by electrophoresis on 4–12% polyacrylamide gels (SDS-PAGE). The gels were treated with Amplify (Amersham Biosciences), and dried for fluorography on a Kodak BioMax MR film (Amersham Biosciences).

### Immunoprecipitations

For immunoprecipitations, 15 μl [^35^S]-labelled *in vitro *transcription/translation products were incubated 2 hours at 4°C with 10 μl rabbit antiserum in a 1 ml final volume of 150 mM NaCl, 10 mM Tris, 1 mM EDTA, 0.5 mM EGTA, 1% Triton X-100, 0.5 mM orthovanadate, 0.5 mM PMSF and protease inhibitor cocktail (Roche Diagnostics Belgium, Vilvoorde, Belgium). This was followed by a further one-hour incubation with protein A-Sepharose (128 mg/ml; Amersham Biosciences). After centrifugation, the pellets were washed 3 times in the immunoprecipitation buffer, and suspended in sample buffer for analysis by SDS-PAGE (4–12% gradient) followed by fluorography as stated above.

### Hamster HLTF cDNA sequences

PolyA(+) RNA was extracted with the Micro-FastTrack mRNA isolation kit (Invitrogen) from 5 × 10^6 ^HKT-1097 cells. Reverse transcription was performed with random primers and the MMLV reverse transcriptase (Promega) according to the protocol provided by the manufacturer. The primers used for PCR were 5' TTGCAGACTGTCCAGTATGG 3' (forward) and 5' ACTGGCATACTATAGCTTGG 3' (reverse); amplification was done with *Pfu *DNA polymerase (Promega) in a Mastercycler gradient (Eppendorf) set for 35 cycles (40 sec at 95°C, 40 sec at 50 °C, 55 sec at 74°C). Protruding A's were added to the PCR product with *Taq *DNA polymerase 15 min at 72°C and it was ligated into the *pCR4-TOPO *vector (Invitrogen). Plasmid DNA was extracted from transformed *E. coli *colonies with the Wizard Plus SV Minipreps DNA Purification System (Promega). Sequences were determined by the dideoxynucleotide chain termination method with CEQ DTCS Quick Start Kit and the CEQ2000 sequencer (Beckman-Coulter). Sequences were analyzed on the Belgian EMBL node  with the EMBOSS software package.

Rapid amplification of cDNA 5' end was performed on 250 ng polyA(+) RNA with the FirstChoice RLM-RACE kit (Ambion) according to the manufacturer's protocols. The specific *HLTF *primers used were the following: 5' GCGTTCAGTTGTCATCTG 3' (outer), 5' ACTGGCATACTATAGCTTGG 3' (inner). The products were resolved by electrophoresis on a 1% agarose gel, the larger fragment was extracted from the gel with the Qiaquick Gel extraction kit (Qiagen), cloned and sequenced as detailed above.

### Hamster genomic sequences

Genomic DNA was extracted from hamster liver with the Scil DNA Tissue Kit in the KingFisher (ThermoLabs System) apparatus. 700 ng DNA were amplified by *Pfu *DNA polymerase with primers mapping to exon 2: 5' TTGCAGACTGTCCAGTATGG 3' (forward) and 5' CCCGTGTAATAGCGTAGTCC 3' (reverse). A denaturation step of 3 min at 95°C was followed by 35 PCR cycles of 30 sec at 94°C, 60 sec at 56°C, 60 sec at 72°C. The resulting 166-bp fragment was cloned and sequenced as detailed above.

### Semiquantitative assessment of DES-induced renal tumors

Neoplasms in hamster kidneys were analyzed as described previously [25] and graded according to size and gross morphology. Briefly, three stages were defined on the basis of the following criteria: 1) Tumorous buds: well-defined, small clusters of tumor cells surrounded by normal kidney tissue (5–200 cells); 2) Medium-sized tumors: more important clusters of tumor cells infiltrating normal kidney tissue (approx. diameter of 150 – 600 μm); 3) Large invasive tumors of large diameter extensively infiltrating the kidney (>300.000 μm^2 ^and up to 80 mm^2^). These stages are illustrated in Figures 3 and 4.

### Reagents

All reagents for immunohistochemistry (primary antibodies, conjugated antibodies or F(ab)2 fragments, ABC kit) came from Dakopatts (Glostrup, Denmark) except when stated otherwise.

### Tissue immunohistochemistry

Immediately after sacrifice, kidney samples were fixed by immersion in alcoholic Bouin mixture (Duboscq-Brazil fluid) for 48 h and embedded in paraffin according to standard procedures. Sections of 4–5 μm thickness were cut serially with a Reichert Autocut 2040 microtome and mounted on silane-coated glass slides.

Tissue sections were immunostained following a slightly modified version of the streptavidin-biotin immunoperoxidase method (ABC method). The primary antibodies, anti-HLTFMet1 (ART2) and anti-HLTFMet123 (ASE2), were tested in preliminary studies to determine the optimal dilutions, 1/350 and 1/300 respectively. The sensitivity of the method was increased by microwave pre-treatment of dewaxed sections in 0.01 M citrate buffer (pH 6.0) for 2 × 5 min at a power of 900 W. After microwave treatment, the sections were incubated in 0.4% hydrogen peroxide for 5 min and rinsed in PBS. Thereafter, the sections were successively exposed to avidin (0.1 mg/ml in PBS) and to biotin (0.1 mg/ml in PBS) for 20 min to block the reactivity for endogenous biotin. After rinsing in PBS, the sections were preincubated in 5% normal goat serum in PBS (NGS-PBS) for 20 min and incubated sequentially at room temperature in the following solutions: (1) primary antiserum for 1 hour; (2) biotinylated goat anti-rabbit IgG (diluted 1:50) for 20 min, and (3) ABC complexes for 20 min. Bound peroxidase activity was visualized by incubation with 0.02% 3, 3'-diaminobenzidine – 0.01% H_2_O_2 _in PBS. The sections were finally counterstained with PAS, hemalun and luxol fast blue and mounted in a permanent medium. Study of the distribution S100 protein relative to HLTF was performed on consecutive sections using rabbit polyclonal anti-S100 as a primary antibody and the demonstration method described above.

### Immunofluorescence staining of cultured cells

For cell immunostaining, twelve-well plates containing sterile round glass coverslips (15-mm diameter) were seeded at a density of 5 × 10^3 ^cells/mm^2^. After 3 days of growth, cell cultures were rinsed with Dulbecco's phosphate buffered saline (DPBS) and fixed at 4°C in 4% paraformaldehyde in DPBS. After 15 min. the fixative was replaced by DPBS where cells were kept at 4°C until immunostaining. For the simultaneous demonstration of vimentin and HLTF, the cells were incubated with a mixture of mouse monoclonal anti-porcine vimentin and rabbit polyclonal anti-HLTF antibodies (ASE2 or ART2). This was followed by exposure to a mixture of fluorescein isothiocyanate (FITC)-conjugated goat anti-mouse immunoglobulins antibodies (F(ab)2 fragments) and biotinylated swine anti-rabbit immunoglobulins antibodies. HLTF-containing immunocomplexes were finally demonstrated by incubating cell preparations with Texas Red-conjugated streptavidin (Pierce, Rockford, ILL, USA). For the simultaneous demonstration of lamin and HLTF, cell preparations were first exposed to anti-HLTF antibodies (ASE2 or ART2), followed by FITC-conjugated swine anti-rabbit immunoglobulins antibodies (Vector Laboratories, Burlingame, CA). The next step consisted in an incubation in presence of rabbit immunoglobulins in order to saturate free binding sites of anti-rabbit immunoglobulins antibodies. Thereafter, cells were exposed to goat polyclonal anti-lamin B antibodies (Santa Cruz Biotechnology, Santa Cruz, CA), followed by biotinylated rabbit anti-goat immunoglobulins antibodies. Finally, lamin-containing immunocomplexes were demonstrated by using Texas-Red-conjugated streptavidin as described above. The preparations were mounted in Vectashield mounting medium (Vector Laboratories, Inc., Burlingame, CA). Immunostained sections were examined on a Leitz Orthoplan fluorescence microscope (Ploem system) and the positive fields recorded with a digital camera (Leica DC 300F).

Controls for the specificity of immunolabeling included the omission of the primary antibody or the substitution of non-immune sera for the primary antibodies. The specificity of anti-HLTF immunostainings was also checked with primary antibodies previously incubated with the corresponding synthetic peptide used as antigen. In each case these controls were negative.

## Results

### Characterization of the antisera

Two human HLTF variants are expressed from the same open reading frame and only differ by the translation start site (Met1 or Met123) [1]. In order to raise an antiserum (ART2) specific for the HLTFMet1 variant, we chose a putative immunogenic peptide i.e. residues 42–56 (VIPPDDFLTSDEEVD) in the amino-terminal sequence missing in the shorter variant. The HLTFMet123 sequence being entirely included in the longer variant, we reasoned that its amino-terminal region might differ in terms of conformation from the corresponding sequence in HLTFMet1. Indeed structure predictions indicated that a set of four putative alpha helices were disrupted by the NH2-truncation in the shorter variant. Aiming to prepare an antiserum (ASE2) specific for HLTFMet123, we thus selected a putative immunogenic peptide corresponding to residues 152 to 166 (GKEENRKAVSDQLKK), surmising that it might be accessible in the short variant but hidden into the HLTFMet1 conformation, at least in non-denaturing conditions. In addition the above mentioned peptides belong to the HLTF DNA binding domain that is highly conserved in the available mammalian sequences (GenBank accession numbers: mouse:#AF165911, rabbit: #U66564 and human: #AJ418064).

**Figure 1:**
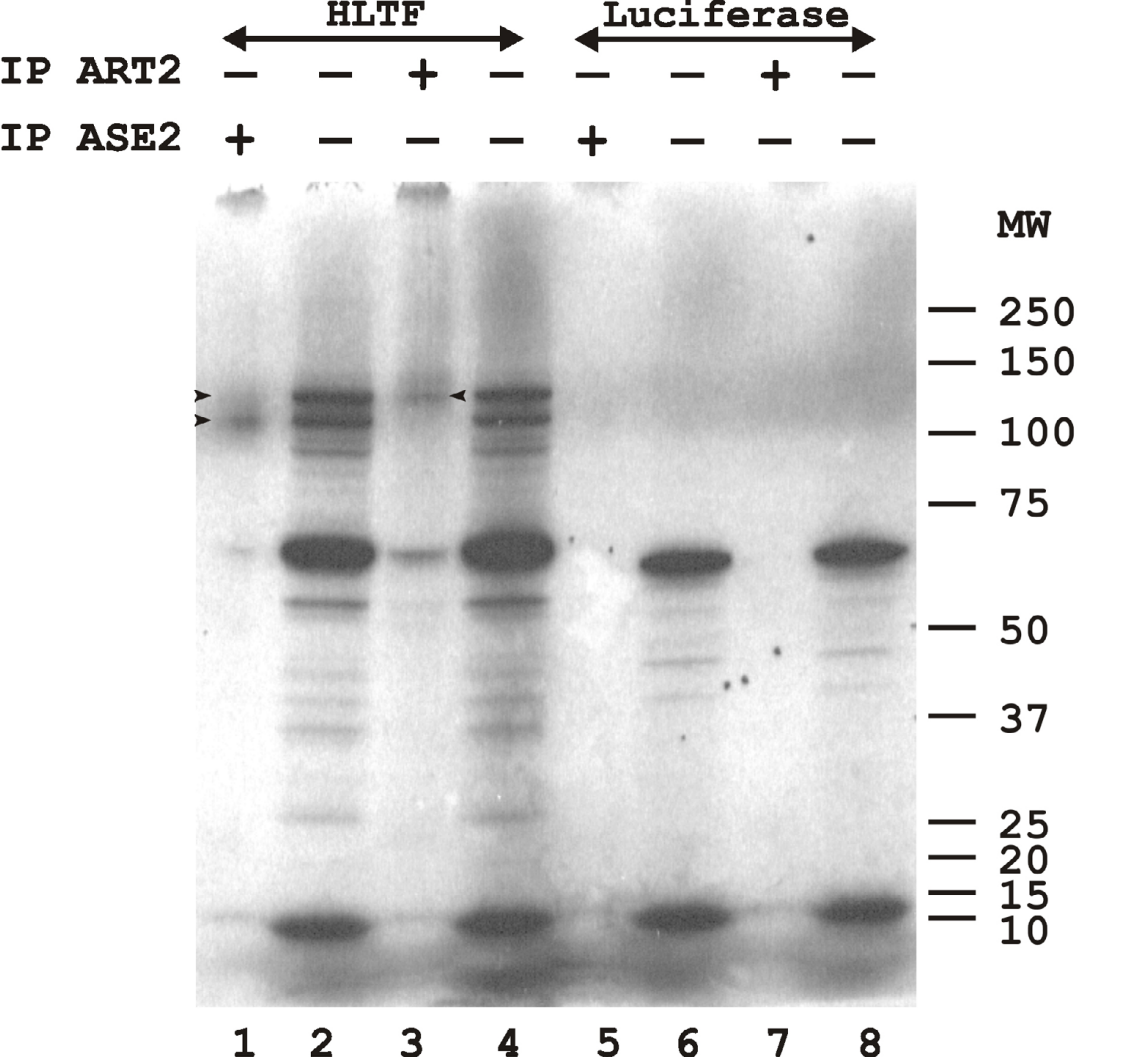
*In vitro *transcription/translation was performed in the presence of [^35^S] cysteine in a TNT rabbit reticulocyte lysate programmed with either *pGEM-4Z-HLTF *encoding both HLTFMet1 and Met123 variants (lanes 1–4) or Luciferase T7 control plasmid (lanes 5–8). The products were resolved by electrophoresis on a 4–12% SDS-PAGE gradient and detected by autoradiography. The samples were either 1,5 μl of total *in vitro *translation products (lanes 2, 4, 6, and 8), or 15 μl of *in vitro *translation products immunoprecipitated with either the ASE2 antiserum directed against HLTFMet123 (lanes 1 and 5) or the ART2 antiserum directed against HLTFMet1 (lanes 3 and 7). The autoradiography was exposed 3 days. The sizes of the molecular weight markers (MW) are given in kDa. Arrowheads indicate the HLTF proteins.

The specificity of these antisera was checked by immunoprecipitation of the human HLTF variants expressed by transcription/translation in a rabbit reticulocyte lysate in the presence of [^35^S] cysteine. The HLTF cDNA template (*pGEM-4Z-HLTF;*[1]) drove synthesis of the two protein variants with apparent molecular weights of 110 (HLTFMet123) and 130 kDa (HLTFMet1) on SDS-PAGE, together with incomplete translation products (Figure 1, lanes 2 and 4). As expected, the ART2 antiserum reacted specifically with HLTFMet1 (lane 3). The ASE2 antiserum immunoprecipitated HLTFMet123 together with a small amount of the larger variant (lane 1): this was not considered sufficient to affect serum specificity in subsequent experiments. Neither antiserum reacted with the negative control, i.e. *in vitro *translated luciferase (lanes 5–8).

### Sequence of HLTF in the hamster

**Figure 2:**
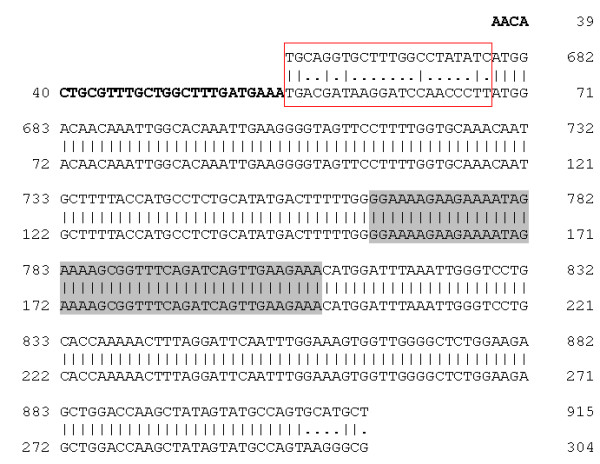
Alignment of the hamster and human HLTF cDNA sequences. A rapid amplification of cDNA 5' end (5' RACE) was performed on polyA(+) RNA extracted from hamster skeletal muscle: the product was cloned and its sequence is given in the top line. The segment in bold corresponds to the adaptor used in the 5' RACE. The human cDNA sequence is a fragment of GenBank #Z46606, with the same numbering (657 to 915). The cDNA sequences are identical over the entire length except for the 22-bp segment boxed in red. The region encoding the HLTFMet123-specific peptide is highlighted in grey.

We wanted to check whether the human peptides used to raise the antisera described above were conserved in the hamster HLTF sequence. We surmised that the 5' part of the HLTF cDNA encoding the DNA binding domain was also well conserved in the hamster, and designed primers for reverse transcription and amplification by PCR in this region based on an alignment of the mouse, rabbit and human cDNA sequences with the Matcher software [34] from the EMBOSS software package. RT-PCR was performed on polyA(+) RNA extracted from HKT-1097 cells, the product was cloned into the *pCR4 *vector, and its sequence was determined. Surprisingly, a 100% identity with human HLTF was found over the 550 bp fragment, suggesting a sample contamination. The experiment was repeated on RNA extracted from hamster liver or testis, confirming the sequence. Two other approaches were used to demonstrate that no human cDNA contamination had occurred. We first determined the sequence of a genomic fragment amplified by PCR within exon 2 and it was again 100% identical to human, confirming the deduced peptide sequence; a similar experiment could not be performed for exon 4 because of its short size. We then cloned cDNA's extending upstream from the second peptide coding region by a 5' RACE approach: products analyzed were found to have 100% identity to the human cDNA excepted for a 22-bp segment that was clearly different (Figure 2). In conclusion, both antisera developed against peptides of human HLTF could be used in the hamster cancer model.

**Figure 3:**
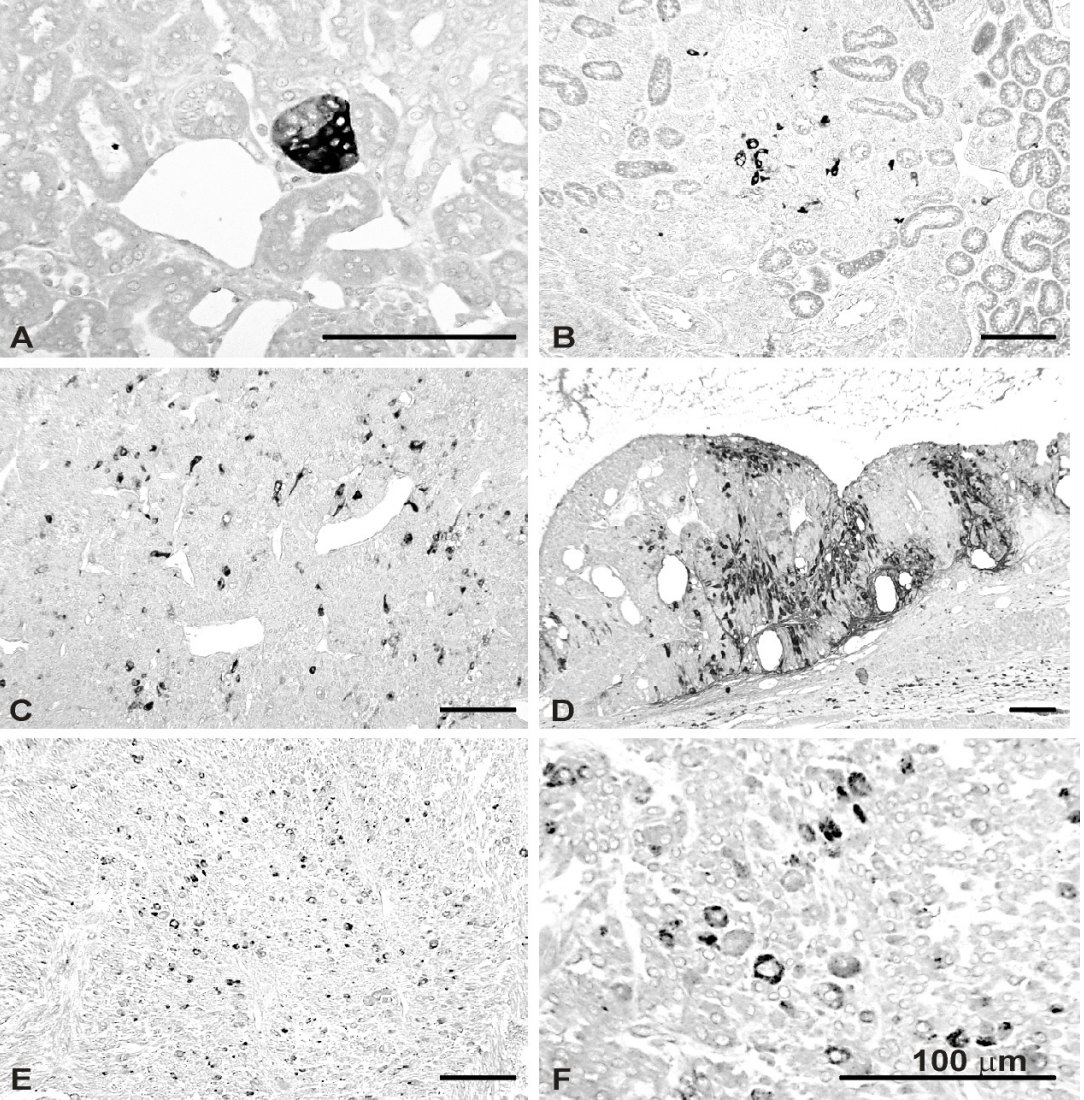
Immunoperoxidase staining of HLTF in kidney and tumor tissue. A-D, kidneys from DES-treated hamsters; E-F, HKT-1097-derived tumor xenograft. Tissue sections were processed for HLTF immunostaining with antisera directed against either HLTFMet123 (ASE2; A, B, D) or HLTFMet1 (ART2; C, E, F). A, HLTF immunolabeling associated with early signs of neoplastic transformation (tumorous bud; two months of DES exposure). B, C, scattered HLTF-positive cells in large renal tumors (6 months of DES exposure). D, cell populations with HLTF immunoreactivity in a large tumor (11 months of DES exposure). E, F, scattered HLTF-positive cells in subcutaneous tumor xenograft.

**Figure 4:**
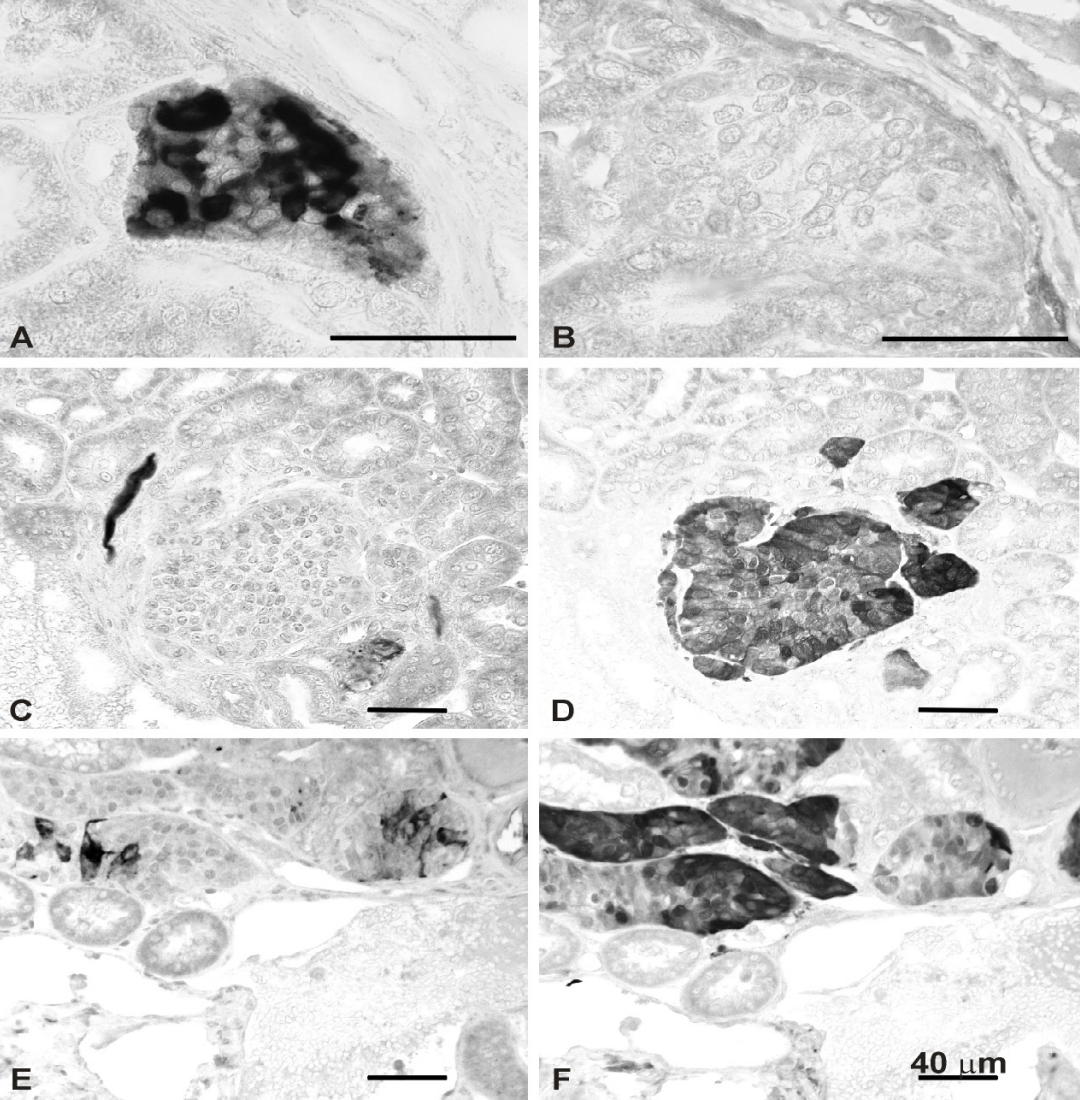
Combined immunolocalization of HLTF (A, C, E) and S100 protein (B, D, F) in DES-induced renal tumors. Immunostaining was performed with the ASE2 antiserum directed against HLTFMet123. A-B, C-D, E-F are corresponding fields in consecutive tissue sections. A-B, a cluster of neoplastic cells with HLTF immunoreactivity (A) appears S100 negative (B) (7 months of DES exposure). C-D, a small tumor negative for HLTF (C) exhibits S100 immunoreactivity (D) (7 months of DES exposure). E-F, mixed population of HLTF-positive (E) and S100-positive (F) cells in a medium size tumor (6 months of DES exposure).

### Morphological observations

In a previous study, some of us have shown that the S100 protein was an early and specific marker of cell transformation related to DES tumorigenicity in the kidney, appearing as soon as 4 months after beginning of drug exposure [25]. Thus, kidneys of animals sacrificed at different time points during DES treatment were processed for S100 as well as HLTF immunostaining. No morphological evidence of HLTF expression was noted in the renal cortex of control animals, but a few collecting tubules were found HLTF positive in the inner medulla of some individuals. This point was not pursued further. Kidneys of hamsters treated with DES for one month still appeared negative for both HLTF and S100. A first evidence of immunoreactive HLTF associated with early signs of neoplastic transformation was seen in the renal tissue of an animal sacrificed two months after beginning of exposure (Figure 3A). Kidneys of this individual and of the other animals of the same group were still S100 negative. HLTF positive cells were a consistent finding in medium size and large tumors of animals treated with DES for 6 months or more. HLTF expression either occurred in scattered cells (Figure 3B–C,), or was seen in tumor cell groups (Figure 3D). Of note, with regard to immunostaining intensity and distribution, no difference was observed between the ASE2 and ART2 antisera. As specified above, the appearance of S100 immunoreactivity was posterior to that of HLTF since S100 positive cells were only seen after four months of DES exposure (not shown). Later on, S100 and HLTF immunoreactivities were not systematically associated. Tumor cell populations could be HLTF positive and S100 negative (Figure 4A–B,), HLTF negative and S100 positive (Figure 4C–D,) or could exhibit both markers (Figure 4E–F,). In the latter case, however, we did not find clear-cut evidence of colocalization (i.e. HLTF and S100 immunoreactivities occurred in distinct cells).

**Figure 5:**
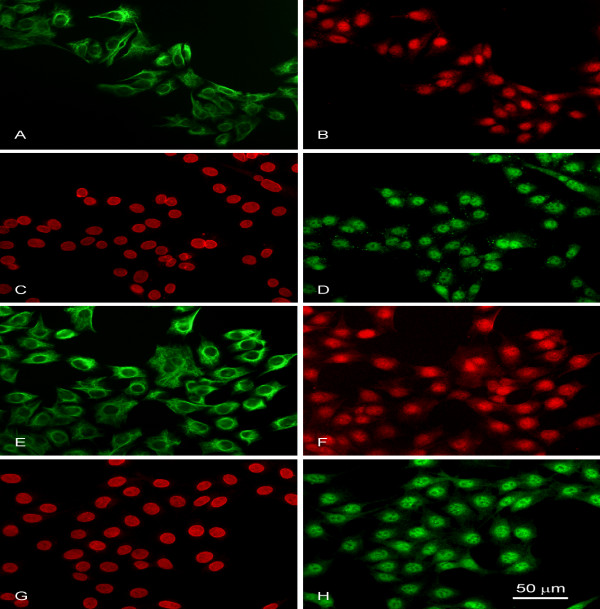
Immunofluorescence staining of HLTF in renal tumor-derived HKT-1097 cells. Cultured cells were processed for double immunofluorescence staining as described in Materials and methods. A, E: general cell appearance revealed by vimentin immunostaining (FITC immunolabeling); B, F: virtually all cells in the microscopic fields display HLTF immunoreactivity as shown by application of antisera (Texas Red labeling) directed against either HLTFMet123 (ASE2; B) or HLTFMet1 (ART2; F). C, G: cell nuclei immunolabeled with anti-lamin B serum (Texas Red labeling); D, H: use of ASE2 (D) or ART2 (H) (FITC labeling) reveals the nuclear localization of immunoreactive HLTF variants.

As shown previously, the HKT-1097 cell line derived from DES-induced renal tumors shares a variety of markers with the neoplasms of origin [29,30]. Immunofluorescence staining clearly demonstrated HLTF expression in HKT-1097 cells (Figure 5). Simultaneous detection of vimentin (an intermediate filament constitutively expressed by HKT-1097 cells) and HLTF by double label immunofluorescence revealed an equivalent level of HLTF expression (as inferred from signal intensity) in all cells (Figure 5A–B, E–F). Besides, combined detection of lamin B (an intermediate filament associated with the nuclear envelope) and HLTF unequivocally localized the latter protein in cell nuclei (Figure. 5c–d, g–h). Similar immunofluorescence patterns were found with the ASE2 (Figure 5B, D) and ART2 antisera (Figure 5G, F), although the signal periphery appeared less sharp with the latter, suggesting a more heterogeneous distribution of the HLTFMet1 protein.

The tumorigenic potential of the HKT-1097 cell line has been demonstrated by its ability to give rise to tumor xenografts in nude mice. As illustrated in Figure 3(E,F), HLTF immunostaining in HKT-1097-derived neoplasm revealed the presence of scattered positive cells throughout the tumor tissue. This suggests that HLTF expression persists for many generations in subpopulations of tumor cells.

## Discussion

Genetic studies in human and mouse have shown that inactivating mutations or deletions of genes encoding subunits of the SWI/SNF complex were associated with cancer, pointing these genes as putative tumor suppressors. In keeping with this idea, inhibition of *HLTF *gene expression caused by promoter hypermethylation was recently observed in 30–70% of human colon cancers and human gastric carcinoma [19,20]. The data that we are presenting here on an animal model of kidney carcinogenesis are in sharp contrast with previously published observations. In fact, in the very study where *HLTF *was initially found to be inactivated in a number of colon and gastric cancers, neither *HLTF *promoter hypermethylation nor loss of expression was found in cancers of other organs such as the breast and the lung [20]. Moreover a 20-fold increase in HLTF mRNA was observed in transformed cell lines derived from various tissues such as breast, ovary, cervix...[11]. We confirmed ourselves these data at the protein level since we showed by Western blot high levels of HLTFMet1 and HLTFMet123 in HeLa (cervix adenocarcinoma) and TE671 (rhabdomyosarcoma) cells (data not shown).

Intriguingly, *HLTF *over-expression was never detected in differential gene expression profiles performed between tumor tissue and its normal counterpart. An explanation might be found in the HLTF expression pattern that we observed here during kidney tumor progression: although every single cell stained for HLTF in tumor buds, the HLTF signal was only detected in about 10% of cells scattered in larger tumors. If a similar pattern occurred in tumors derived from other tissues, the 10–20 fold increase in HLTF expression in 10% of tumor cells would be diluted out in the population of HLTF negative cells, and it might not be detected upon analyses of whole tumor homogenates. This hypothesis will have to be evaluated by analyses of HLTF expression in clinical samples of early primary tumours.

Preliminary observations have also shown on a model of rat kidney regeneration that HLTF was expressed very transiently (12–24 h) during the late steps of tubular cell differentiation. (unpublished data). This restricted period of expression associated with an asynchronous development of tumoral foci might explain why only 10% of tumor cells exhibit HLTF overexpression. An alternative explanation is suggested by a recent work [35] indicating that a small population of cells endowed with unique self-renewal properties and tumorigenic potential is present in tumors. According to this model, if HLTF is only expressed by these cancer stem cells and their direct progeny, positive cells should appear randomly distributed within kidney tumors.

In the present study, we have detected HLTF protein expression at early stages of development of renal tumors induced by DES in Syrian golden hamsters. The protein was detected before the appearance of S100, an early tumor marker previously identified in this model [25] : indeed two months after initiating DES administration, we already found tumor buds consisting of only a few dozen cells that all exhibited strong immunostaining with the antisera against HLTF. These observations also suggest that HLTF is a precocious marker expressed either simultaneously with or even before other known markers of estrogen-induced kidney tumours such as ER, PR [36], c-MYC, c-FOS [37] or cyclins E, D1 [38].

Some of us have recently used an anti-galectin-1 antibody for immunohistochemical staining in the same cancer model, and detected small clusters of presumably preneoplastic cells at the corticomedullary junction already one week after starting DES administration. After one month of exposure, tumorous buds positive for both galectin-1 and 3 were detected at the same location, in the perivascular connective tissue next to large blood vessels [39]. These data suggested that these galectins might be involved in the early carcinogenesis stages of this model. Intriguingly, such an implication was also suggested in human colon cancer [40] where HLTF is inactivated by hypermethylation of its promoter. HLTF (this study) as well as galectin-1 and 3 [39] are expressed in every cell of the established HKT-1097 line grown *in vitro*, raising the possibility that HLTF might activate galectin-1 and -3 gene expression. It will thus be very interesting to evaluate whether the same cells might express these proteins in the hamster kidney cancer model by co-immunostaining with galectin-1 and 3 at various stages of tumor development, including very early stages of DES treatment when only galectin-1 is detected.

Finally, converging information suggest that the estrogen-induced tumors could arise from germinal interstitial stem cells [24,36] or from an unidentified precursor cell associated to the sheath of Schwann cells [25]. These observations thus suggest that HLTF could be a marker of undifferentiated cells and not directly related to the mechanisms of carcinogenesis. Yet, previous observations of HLTF expression in mice embryos [11] and in a model of rat kidney regeneration (unpublished data) have shown that HLTF is detected very late in the course of tissue differentiation.

We observed a rarefaction and a scattered distribution of HLTF positive cells during tumor progression. Several reasons could be put forward to explain those data. First, the few cells that form the tumoral bud might evolve to form different clonal populations showing different phenotypic drifts. Indeed, some of us have shown that progression of DES-induced renal tumor from tumor buds to advanced malignancies was associated with phenotypic variation and clonal divergence of neoplastic cells [41]. Another hypothesis is that HLTF over-expression might be involved in early steps of cellular transformation, and could later on be replaced by a more efficient survival strategy consisting of genetic alterations that would confer higher proliferative/invasive capacity. Indeed, HLTF expression did not seem linked to proliferation since HLTF-positive cells and proliferating cells (BrdU staining, not shown) belonged to distinct subpopulations. Moreover, if all cells were HLTF positive in HKT-1097 cells cultured *in *vitro, the same decrease in HLTF signal was observed in tumours derived from these cells in nude mice (Fig 3E–F,). Other studies have indicated that HLTF expression was coupled to terminal differentiation in mouse development [11]: one could imagine that even if HLTF over-expression were to favor immortalization, it might still slow down proliferation. In this line of reasoning, during tumor progression, tumor cells might circumvent the antiproliferative effect of *HLTF *gene by hypermethylation, as observed in advanced colon adenomas [20]. The observation that some HLTF-positive cells always remain present during progression and are found scattered in the tumor mass is suggestive of a paracrine interaction: HLTF-positive cells might stimulate proliferation of the surrounding HLTF-negative cells by secretion of different growth factors.

There is also ample information showing that estrogen-induced aneuploidy is detected in both early tumor foci and in primary tumors of the kidney [36]. This chromosomal instability seems to be elicited in kidney multipotent interterstitial stem cells by an overexpression of c-*myc*, subsequent to estrogen administration [42]. Currently, the involvement of HLTF in this estrogen-induced mechanism has not been fully explored but preliminary observations suggest that HLTF could play a role in generation of aneuploidy. Indeed, we have detected, by using a yeast two-hybrid screen with an HLTF domain as the bait (unpublished observations), a cDNA encoding the kinetochore protein (CENPE) [43]. This suggests a potential involvement of HLTF in the movement of mammalian chromosomes and in spindle elongation.

One of the rationale behind the present study was that the *HLTF *gene encoded two protein variants with different biological functions, only the shorter of which (HLTFMet123) presented a transcriptional activity [1,13]. In the present study, we wondered whether an imbalance in the variant ratio might be linked to transformation. However no difference could be found in the immunostaining patterns demonstrated by the specific antisera (ART2 and ASE2) raised against the individual variants. A word of caution should be given here to rule out a putative artifact. The immunogenic peptide "specific" for HLTFMet123 is also present in HLTFMet1, and the specificity of the ASE2 serum only relies on the three-dimensional structure of the larger amino-terminal domain in HLTFMet1 where the sequence of the peptide used to raise ASE2 is buried. One could argue that the microwave pre-treatment of renal sections performed for antigen retrieval could have denatured the HLTF proteins and expose this immunogenic peptide, allowing recognition of the HLTFMet1 protein by the antiserum "specific" of HLTFMet123. However, the immunofluorescence staining of HKT-1097 cells did not involve such harsh treatment and anyway revealed very similar staining patterns for both antisera, with just a fuzzier HLTFMet1 staining at the nuclear periphery as compared to HLTFMet123 (Figure 5B, D*vs *Figure 5F, H). Interestingly the HLTF RING domain has been found to interact with an atypical Type IV P-type ATPase, a putative phospholipid pump located at the inner nuclear membrane [44].

The HLTF proteins were previously shown to locate in the nucleoplasm of HeLa cells, as expected for SWI/SNF proteins [1]. In the present study, a nuclear localization was also clearly observed in HKT-1097 cells fixed in paraformaldehyde. In contrast, in the kidney tumors fixed in alcoholic Bouin's mixture, most HLTF-positive cells presented a strong cytoplasmic signal, while only few cells displayed a nuclear staining. Again, the different locations observed between cells in culture and tumoral tissues might be artefactual and caused by tissue processing (fixation, dehydratation, dewaxing) and/or the treatment applied for antigen retrieval. Alcoholic Bouin's mixture was not used for HKT-1097 cells, but experiments using another alcoholic fixation method (Carnoy) on these cells indeed showed a significant alteration of the nuclear signal (data not shown). However, in a few tumor samples that were fixed in paraformaldehyde we could detect the same proportion of HLTF-positive cells with strong cytosolic staining as found in the present study (data not shown), suggesting other explanations than processing artefacts. A similar conclusion was drawn in a previous study on the p53 oncoprotein [45]. It showed that the cytoplasmic distribution of TP53 was genuine and not the result of pre-treatments like poor fixation or antigen unmasking, and that the latter actually improved the quality of the immunocytochemical reaction [45]. Several examples are known of transcription factors or associated cofactors that shuttle between the nucleus and the cytoplasm according to various stimuli. Sequestration in a cellular compartment is indeed a way to regulate transcription activity. NFκB is a classical example: it is kept inactive as a cytoplasmic complex with IκB until the latter undergoes phosphorylation and proteosomal degradation, releasing NFκB that migrates to the nucleus for transcription activation (reviewed by [46]). Conversely, MEF2 is sequestered in an inactive nuclear complex with a histone deacetylase that is translocated to the cytoplasm upon phosphorylation by a calcium-dependent kinase, allowing MEF2 to activate transcription of genes involved in terminal muscle differentiation [47]. One could hypothesize that sequestration of HLTF outside of the nucleus might be part of the transformed phenotype as shown previously for p27Kip, a negative regulator of the cell cycle that accumulates in the cytoplasm of colorectal tumors [48].

## Conclusion

In conclusion, our data suggest that HLTF protein expression is linked to early transformation in a model of hormonal carcinogenesis and that HLTF protein detection might be of value as an early marker in some human cancers.

## Competing interests

AB, CG, DN, FR, GD, GL, GT, MW and OL have applied for a patent whose value may be affected by this publication.

## Authors' contributions

AB is the promoter of the HLTF project. CG performed the transcription/translation and immunoprecipitation experiments. DN and GT carried out the hamster carcinogenesis experiments and performed immunohistochemical analysis of tumor samples. FJ produced the mouse tumor xenografts. FR and MW performed the immunohistochemical staining of tumor samples and the immunofluorescence on cultured cells. GD performed the RNA and DNA study, validated the anti-HLTF antibodies in the hamster model and finalized the manuscript. DN and GL developed immunofluorescence procedures and supervised this analysis on cultured cells. AB, CG, FR, GD, GL and GT collaborated to the drafting of the manuscript and the critical review of its content. OL produced the HLTF antibodies and provided expertise for their validation.

All authors read and approved the final version of the manuscript.
